# Metabolism Plays a Key Role during Macrophage Activation

**DOI:** 10.1155/2018/2426138

**Published:** 2018-12-10

**Authors:** Marion I. Stunault, Gaël Bories, Rodolphe R. Guinamard, Stoyan Ivanov

**Affiliations:** Centre Méditerranéen de Médecine Moléculaire-Université Côte d'Azur-INSERM U1065, Team 13, France

## Abstract

Monocyte and macrophage diversity is evidenced by the modulation of cell surface markers and differential production of soluble mediators. These immune cells play key roles in controlling tissue homeostasis, infections, and excessive inflammation. Macrophages remove dead cells in a process named efferocytosis, contributing to the healthy tissue maintenance. Recently, it became clear that the main macrophage functions are under metabolic control. Modulation of glucose, fatty acid, and amino acid metabolism is associated with various macrophage activations in response to external stimuli. Deciphering these metabolic pathways provided critical information about macrophage functions.

## 1. Introduction

Monocytes and macrophages are part of the mononuclear phagocytic system and share multiple cell surface markers. Historically, monocytes were described as necessary precursors for tissue-resident macrophages. However, this concept has recently been challenged by the discovery of embryonically derived macrophage subsets in several tissues including those of the brain and heart [1–5]. The pool of embryonically derived macrophages renews via self-proliferation, independently from the pool of bone marrow-generated monocytes [6]. Another observation that defied the initial concept is the presence of monocytes in peripheral tissues, outside the blood vasculature [7]. A genome-wide transcriptional analysis of macrophages, isolated from a large pattern of tissues, revealed that each subset of tissue-resident macrophages has a unique transcriptional profile despite a conserved core signature. This suggests diversity in the pool of tissue-resident macrophages driven by their local environment [8]. Those observations were followed by the identification of transcription factors controlling the development and fate of a single population of tissue-resident macrophages [9–12]. For instance, tissue-specific signals induce a core signature refinement by signal-dependent transcription factors [13]. This allows macrophages to perform unique functions. For example, splenic macrophages are dependent on the transcription factor Spi-C regulated by heme concentrations. Red pulp splenic macrophage absence in Spi-C-deficient mice induces splenic iron accumulation due to impaired erythrocyte clearance [14]. Another example is the large peritoneal macrophage population that relies on dietary retinoic acid induction of the transcription factor Gata6 to develop and survive [11]. Macrophage plasticity with respect to environmental cues is supported by large peritoneal macrophage loss of phenotypic and transcriptional core-specific signature once transferred to a new environment or when Gata6 is genetically removed [11, 15]. However, comprehensive links between local microenvironmental cues and tissue-resident macrophage fate are still largely lacking.

Recently, macrophage modulation of metabolism emerged as a central player during their activation [16]. Nevertheless, to what extent does the wide transcriptional diversity of tissue-resident macrophages reflect on and to what extent is it driven by environmental and metabolic adaptations remain to be elucidated. The purpose of this manuscript is to review the metabolic demands of monocytes and tissue-resident macrophages.

## 2. Mononuclear Phagocytic Cells

### 2.1. Monocytes

Monocytes are generated in the bone marrow from progenitor cells during a process named myelopoiesis (for review, see [17]). Once these cells complete their maturation, they eventually egress to the blood circulation and peripheral tissues. Monocyte retention in the bone marrow and their entry into blood vessels are under the tight control of chemokine and chemokine-receptor interactions. For instance, the CXCR4-CXCL12 and CCR2-CCL2 axes have been implicated in this process [18–20]. The contribution of these pathways is illustrated through blood monocyte drop and bone marrow accumulation in genetic models where these pathways are ablated. Recently, metabolic factors have also been implicated in the control of monocyte pool in the bone marrow [21, 22].

Blood monocytes are easily separated into at least 2 subsets based on the expression of the cell surface marker Ly6C in mice. Using this criterion, classical/inflammatory monocytes are characterized by a high level of Ly6C, while nonclassical/patrolling monocytes are Ly6C^low^. The latter subset is dependent on the transcription factor Nr4a1 (nuclear receptor subfamily 4 group A member 1), as demonstrated by their loss in Nr4a1-deficient mice [23]. Interestingly, Ly6C^low^ monocytes are resident of the blood vasculature where they play a critical role in vessel homeostasis eliminating stressed and dying endothelial cells [24]. Recently, it was also demonstrated that Ly6C^low^ monocytes detect metastatic cells inside blood vessels and, with the help of NK cells, protect mice against metastatic development [25]. As opposed to the resident nature of the Ly6C^low^ population, Ly6C^high^ monocytes leave the blood circulation to enter injury sites. Once more, the CCL2-CCR2 axis is critical for Ly6C^high^ monocyte peripheral tissue recruitment during infection and inflammation [19, 26]. Once monocytes infiltrate peripheral tissues, they can differentiate into inflammatory macrophages.

### 2.2. Macrophages

**Figure 1:**
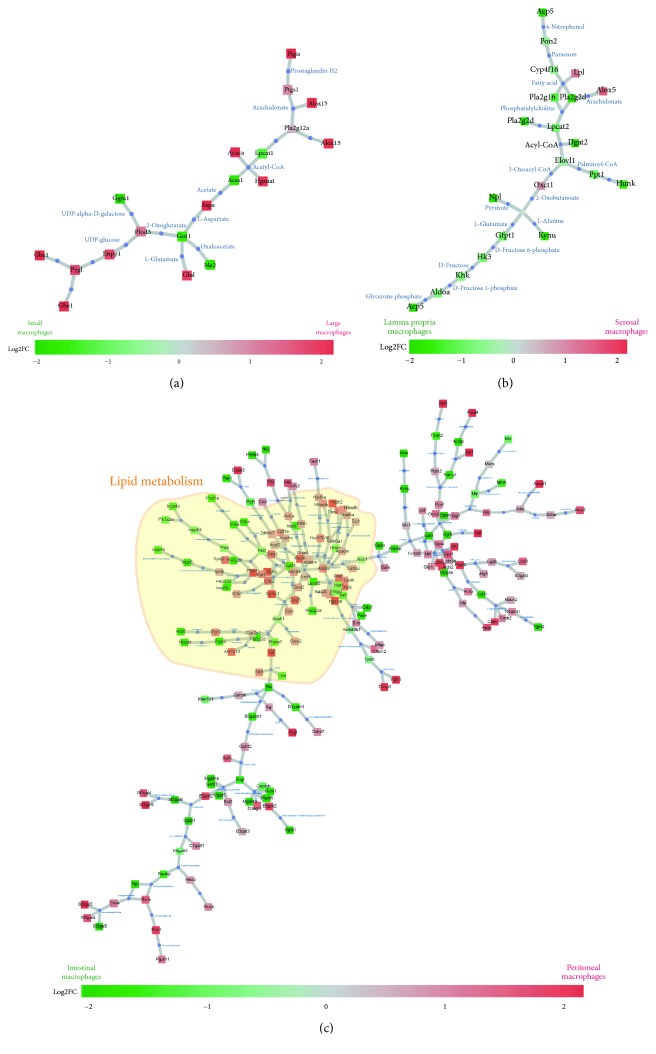
Metabo-transcriptional network representing differences between small and large peritoneal macrophages (a), serosal and lamina propria intestinal macrophages (b), and peritoneal (large and small) versus intestinal (serosal and lamina propria) macrophages (c). The analysis is based on the gene expression data from Immgen Consortium. Boxes are colored according to differential enzyme expression (green versus red) as indicated in the figure panels.

Macrophages are critical players in host defense against infections, during inflammation and in response to injury (for review, see [27]). Macrophages are highly specialized in phagocytosis contributing to tissue remodeling and to the removal of cellular debris. At a steady state, each organ independently regulates the size of its intrinsic macrophage pool. This implies lifelong residency of tissue macrophages and is probably responsible for the distinct transcriptional signature that each tissue macrophage population adopts [15, 28]. Interestingly, this specific signature also includes metabolic traits. A recent study that used metabolic transcriptional signature revealed, for example, that microglia, brain-resident macrophages, seem to have low steady-state metabolic demands [29]. We tried to illustrate the impact of organ cues on the transcriptional signature of metabolic genes by comparing 4 different macrophage populations, of which two populate the peritoneal cavity and the other two populate the intestine (Figure 1). Large and small peritoneal macrophages have a similar metabolic signature despite their different origins and transcriptional dependencies (Figure 1(a)) [9–11, 30]. The same observation is also valid when two distinct populations of intestinal macrophages are compared to each other (Figure 1(b)). Nevertheless, when both peritoneal macrophages were compared to intestinal macrophages altogether, the number of metabolic pathways differentially expressed was much larger suggesting that changes in resources inside different environments could be the main metabolic challenge that tissue macrophages have to adapt to (Figure 1(c)). As it is now suggested that macrophage key functions such as cytokine release and phagocytosis are associated with singular metabolic signature [29], organ cues are probably major drivers of their functions as well. From organ cues and key transcriptional regulators, which prevail in defining macrophage function and responses, are and will be an exciting area of research. As an example in Figure 1 of such complex and recursive regulation, the metabolic differences between small and large peritoneal macrophages seem to be defined by the large peritoneal macrophage mandatory transcription factor GATA6, while GATA6 expression is driven on maturing macrophages by the availability of the key metabolite vitamin A [11]. Interestingly, intestinal macrophages and alveolar macrophages are predicted to rely on inositol and arachidonate pathways, respectively [29]. Beside adaptation to tissue cues, macrophage activation is also consubstantial with metabolic rewiring. This is illustrated during macrophage activation with canonical microbial compounds such as LPS (lipopolysaccharide), a specific TLR4 ligand (Toll-like receptor 4), leading to classically activated (M1) proinflammatory macrophage generation. This activation induces an increased glycolysis and a disrupted Krebs cycle, in order to supply cell metabolic adaptations and cytokine production. On the other hand, macrophage stimulation with IL-4 (interleukin 4) generates alternatively activated (M2) anti-inflammatory macrophages. In this case, cells rely on fatty acid oxidation (FAO) and oxidative phosphorylation to support the metabolic program initiated by IL-4.

A metabolic shift observed between macrophages from different tissues suggests that they adapt their metabolism to fit local resources. Metabolic adaptation might also be a way to vary functions and responses starting with the same resources. As an example, Gata6 deficiency beside its mandatory role for maintaining large peritoneal macrophages leads to an increased alternative polarization in the remaining large peritoneal macrophages associated with altered metabolism [9]. Here, we will discuss the metabolic pathways in macrophages at a steady state and during activation.

## 3. Metabolic Control of Monocyte and Macrophage Functions

### 3.1. Glucose

**Figure 2:**
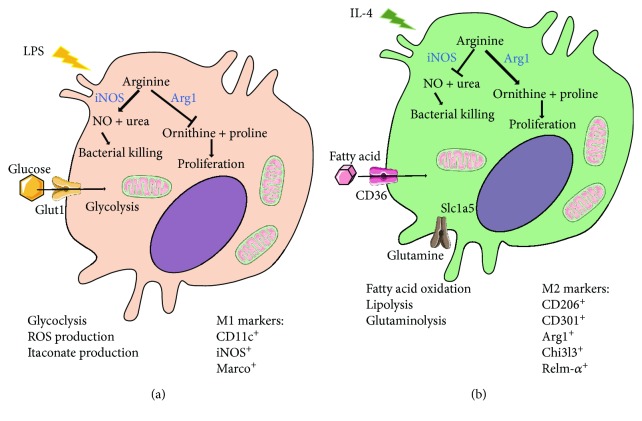
Schematic representation of M1 (a) and M2 (b) macrophage metabolic demands. Common M1 and M2 markers are indicated in (a) and (b), respectively.

The proinflammatory versus anti-inflammatory (M1/M2) macrophage classification has been criticized as an oversimplification of a more complex reality. Indeed, it appears that the local tissue signaling leads to a much broader spectrum of macrophage phenotypes in tissues. How local microenvironment impacts macrophage metabolism and functions is still a field of intense research. M1/M2 classification has nevertheless allowed the demonstration of how metabolic plasticity is associated with and participates in macrophage polarization. A pioneering work demonstrated that LPS stimulation induces increased glucose uptake and glycolysis into macrophages (Figure 2(a)). Members of the Glut family mediate glucose uptake, and macrophages express high levels of Glut1 and Glut3 but lack Glut2 and Glut4 [31]. Among the members of the Glut family, Glut1 expression is upregulated following LPS exposure and is required for increased glucose uptake [31–33]. Furthermore, macrophage-specific Glut1 overexpression is associated with increased glycolysis and pentose phosphate pathway intermediates mirrored by the induction of proinflammatory mediators such as TNF*α* (tumor necrosis factor-alpha) and IL-6 [31]. This is also paralleled by increased ROS production that might drive the proinflammatory signature of Glut1-overexpressing macrophages. Nevertheless, another study documented that glycolysis is crucial for macrophage inflammatory cytokine production, whereas Glut1 overexpression has no detectable effect on this parameter [33]. This study revealed that increasing the macrophage glycolytic rate has no impact on cardiovascular disease and atherosclerosis and in particular on a plaque area [33]. This was quite surprising as macrophages are the main cell population composing the plaque. Recently, the use of 18F-fluorodeoxyglucose- (FDG-) positron emission tomography (PET) imaging, which provides a noninvasive measure of tissue glycolysis, demonstrated increased glucose uptake in plaque areas in cardiovascular disease-affected patients [34–36]. The biological significance of increased glucose incorporation in the atherosclerotic plaque still needs to be identified. One could speculate that this increase just reflects changes in cell populations that normally occupy the vessels or it could be a reflection of the hypoxic nature of plaques that favor glycolysis in multiple cell types. By contrast, in hematopoietic tissues, enhanced glycolytic activity was also detected and could predict the production, differentiation, and activation of immune cells. Glut1 deficiency in hematopoietic precursor cells during atherosclerosis directly affects myelopoiesis and prevents disease outcome [21].

Glucose metabolism is also central during macrophage pathogen phagocytosis [37]. Nevertheless, the macrophage glucose dependence during microbial infections represents an opportunity for pathogens to defy the host immune system. Pathogens, such as *Candida albicans*, consume high amount of glucose and thus limit local glucose availability for immune cells and notably macrophages [38]. This dampens macrophage glucose uptake, decreases their phagocytic skills, and limits their survival during *C. albicans* infection [38]. The extent to which this competition affects infection outcome is an important question to solve in an already complex field. Thus, LPS induces an Hif1*α*-dependent heightening of glycolysis in macrophages. Blocking glucose uptake early during LPS sepsis (in a sterile setup and therefore in the absence of bacterial glucose competition) has been shown to blunt inflammation and sepsis. That glycolysis impact is on macrophages is supported by similar protection and reduction of inflammation when using Hif1*α* macrophage-deficient mice [39]. Nevertheless, inhibition of glycolysis later on, in an otherwise similar setup of LPS-induced sepsis, does not impact inflammation but is still protective. This protection is due to a modulation of nonimmune organ adaptation to stress imposed by inflammatory states, notably in the brain [40]. By contrast, blocking glycolysis is lethal during influenza virus infection [40]. Further analysis revealed that blocking glycolysis does not affect viral burden or immune cell infiltration. Interestingly, inflammation does not seem to account for the effect, and blocking glycolysis did not impact on the lung tissue morphology in virus-infected animals. Blocking glycolysis during flu infection rather decreased critical vital processes such as body temperature and heart rate. With respect to glucose competition between immune cells and pathogens, it is quite fascinating that the lethality of multiple infections might be due to appropriate immune or nonimmune metabolic protection chosen in response to the first infection that was inappropriate and ultimately enhanced the competitive metabolic advantage or destructive power of the second infection. Such scenario could explain excess morbidity associated with influenza infection that is often observed during pneumococcal superinfection.

### 3.2. Fatty Acids

During alternative polarization in response to IL-4 stimulation, macrophages rewire their metabolic demands towards fatty acid oxidation into their mitochondria (Figure 2(b)) [41]. Alternative macrophage polarization relies on the transcription factors Ppar-*γ* (peroxisome proliferator-activated receptor-gamma) and Ppar-*δ*, as well as on their coactivator PGC1*β*, for an efficient metabolic reprogramming [42–44]. Indeed, this program promotes fatty acid oxidation and mitochondrial biogenesis. To fulfill their metabolic needs, alternatively activated macrophages have to generate or internalize fatty acids (FA).

#### 3.2.1. Cell-Intrinsic Fatty Acid Metabolism

Free fatty acids (FFA) are released through lipolysis from triacylglycerol (TG). Lipolysis mostly occurs in adipocytes and participates in triacylglycerol stock mobilization during exercising, fasting, or adrenergic receptor stimulation. Adipocyte TGs are stored inside lipid droplets. The first enzyme that breaks triacylglycerol into diacylglycerol (DG) and a FA is named adipose triglyceride lipase (Atgl), encoded by the *Pnpla2* gene in mice. Secondarily, the hormone-sensitive lipase (Hsl), encoded by the *Lipe* gene, uses DG as a substrate and transforms it into monoacylglycerol (MG) and releases one more FA. The final step during lipolysis is controlled by the monoacylglycerol lipase (Mgl) and by the breaking down of MG into FA and glycerol. Atgl expression is not strictly limited to adipocytes and, to a lesser degree, is also detected in cardiac and skeletal muscle cells, testis, and immune cells such as macrophages and neutrophils [45]. Metabolomic analysis of classically (M1) and alternatively (M2) activated macrophages revealed MG accumulation in M2 cells, suggesting increased lipolysis [41]. Nevertheless, Atgl^−/−^ peritoneal macrophages responded with the same magnitude to IL-4 stimulation and upregulated M2 cell surface markers to a similar extent as Atgl-sufficient control cells [41]. Interestingly, the activity of Lipa (lysosomal acid lipase), another enzyme that uses fatty material as a substrate, is increased in IL-4-stimulated macrophages [46]. Lipa inhibition leads to defective M2 polarization and decreased fatty acid oxidation [41]. Additionally, macrophage deletion of Ascl1 (acyl-CoA synthetase long-chain family member 1), encoding for a key enzyme involved in fatty acid synthesis that catalyzes the thioesterification of fatty acids, is associated with diminished inflammatory cytokine (TNF*α* and IL-1*β*) and chemokine (CCL2) production, pointing out the role of fatty acid synthesis during the proinflammatory response [47]. Altogether, these data demonstrate that macrophage fatty acid metabolism supports the metabolic rewiring in response to external stimuli.

#### 3.2.2. Cell-Extrinsic Fatty Acid Metabolism

Another option for alternatively activated macrophages to supply their metabolic needs is to internalize and incorporate metabolites from the local microenvironment. This requires cell surface receptors or transporters that recognize a specific metabolite combined with intracellular delivery to a precise organelle where this metabolite will eventually be included into macrophage metabolic circuits. During alternative macrophage polarization, it was recently demonstrated that fatty acid uptake via the cell membrane receptor CD36 plays a crucial role for their metabolic adaptation [41]. CD36-dificient macrophages have impaired alternative polarization suggesting that TG uptake and delivery into lysosomes, the place where Lipa is located, play an essential role in M2 polarization. However, macrophages deficient in Ldl receptor (Ldlr^−/−^) do not have impaired alternative polarization suggesting that this receptor is not required for fatty acid uptake [41]. Finally, as much as 3 × 10^8^ cells in our body die by apoptosis each hour, and CD36 together with integrins and the TAM/opsonin system participates in the uptake of apoptotic cells by tissue macrophages representing a tremendous source of metabolite input at a steady state and during apoptotic bursts associated with development or inflammation. This is strongly associated with metabolic changes in macrophages. For example, it has been shown that efferocytosis triggers mitochondrial uncoupling trough the increased expression of uncoupling protein 2 (UCP2) [48]. Furthermore, an increase in FAO is also observed in macrophages during efferocytosis, which could be due to the excess of lipids brought by dead cell membranes [48]. Interestingly, an excess of glucose [48, 49] or changes in the environment modulating macrophage metabolism like inflammation [50] and oxidative stress [51] as well as oxidized LDL [52] and hypoxia [53] inhibit efferocytosis capacity, suggesting that the macrophage metabolic profile is intrinsically linked to its function [48, 49]. Moreover, it is well known that M2 macrophages are more efficient than M1 macrophages to perform efferocytosis, suggesting that different metabolic rewiring could determine the efferocytosis ability in macrophages [50, 54]. Efferocytosis, beside its metabolic input, directly regulates macrophage polarization toward an anti-inflammatory and prohomeostatic phenotype. This includes transcriptomic upregulation of lipid digestive and secretive capacity. Optimal efferocytosis also indirectly prevents inflammatory signals triggered by noningested apoptotic cells. Lipid efflux might also insure redistribution of lipids systemically via lipoprotein export and/or to the local tissue from which the apoptotic cell comes from.

### 3.3. Itaconate

In M1 macrophages, the TCA cycle is interrupted at two points. The first breaking point occurs at the level of isocitrate dehydrogenase 1 (Idh1), which is strongly inhibited in LPS-stimulated macrophages [55]. As a consequence, *α*-ketoglutarate levels are reduced whereas (iso)citrate accumulates and serves as a precursor for itaconate synthesis. Itaconate is an antibacterial metabolite which is among the most highly induced metabolites in activated macrophages [55, 56]. Itaconate controls the second breakpoint by inhibiting succinate dehydrogenase (Sdh), which mediates oxidation of succinate into fumarate [55, 56]. As a consequence, malate accumulates, which is explained by the induction of the aspartate argininosuccinate shunt [55], which happens to be essential for the increase in nitric oxide (NO) production by activated macrophages [55]. Itaconate activity results in succinate accumulation and decreased oxygen consumption [56–58]. Surprisingly, itaconate exerts anti-inflammatory effects by limiting IL-1*β*, IL-18, IL-6, and IL-12 expression and NO production [56, 58]. In mice that are deficient in aconitate decarboxylase 1 (Irg1), the enzyme which converts (iso)citrate into itaconate, an increase in mitochondrial respiration, a decrease in succinate accumulation due to an increase in its conversion into fumarate and malate, and an increase in proinflammatory gene and NO production are observed [56]. Thus, itaconate appears to be one of the master regulators of metabolic reprogramming and inflammation in macrophages [56].

Type I interferons, typically secreted during viral infection, induce Irg1 expression in macrophages and subsequent itaconate production [59]. The newly generated itaconate inhibits type I interferon immune response suggesting the existence of a negative feedback loop orchestrated by this compound. Recently, it was suggested that itaconate also activates the transcription factor Nrf2 (nuclear factor (erythroid-derived 2)-like 2), a key element of the anti-inflammatory response in macrophages [59]. Nrf2 activation follows itaconate alkylation of cysteine residues of KEAP1 (Kelch-like ECH-associated protein 1), a protein that normally associates and promotes the degradation of Nrf2. Nevertheless, it was elegantly demonstrated that itaconate additionally induces electrophilic stress targeting glutathione levels and affecting cellular oxidative stress. Itaconate inhibited late, but not initial, transcriptional response to LPS. One of these early responses includes the transcription factor I*κ*B*ζ* which is the major orchestrator of the secondary transcriptional response. Thus, itaconate did not affect I*κ*B*ζ* mRNA induction but affect I*κ*B*ζ* protein levels and the subsequent secondary response [60]. Interestingly, I*κ*B*ζ* protein regulation is Nrf2-independent and relies on the transcription factor ATF3 (activating transcription factor 3) [60]. Importantly, in vivo administration of cell-permeable derivatives of itaconate protects mice against psoriasis when challenged with a TLR7 agonist, demonstrating a novel potential therapeutic opportunity [60]. Further, myeloid cell-specific Irg1-deficient mice challenged with Mycobacterium tuberculosis (Mtb) showed an excessive accumulation of neutrophils associated with decreased survival rate [61]. This shows that Irg1 and endogenous itaconate play an important role in dampening inflammation during Mtb lung infection. Irg1 and itaconate are also induced in peritoneal macrophages in tumor-bearing mice [62]. Metabolic reprogramming of peritoneal macrophages and increased OXPHOS parallels this observation. Irg1 inhibition in peritoneal macrophages decreases tumor progression demonstrating that this pathway could also be of therapeutic interest in cancer [62]. In humans, branched-chain amino acid (BCAA) catabolism has been shown to be involved in the IRG1/itaconate axis in activated macrophages [63]. In mice, branched-chain aminotransferase 1 (BCAT1) inhibition through the leucine analogue ERG240 administration decreases itaconate production and macrophage infiltration in nephrotic nephritis models pointing out to amino acids as participants in the pivotal itaconate control of macrophage function [63].

### 3.4. Amino Acids

Amino acids are key elements in immune cells for building proteins and nucleotides. They are also critical metabolic intermediates that participate in a variety of physiological processes [64]. A seminal work during the early 1980s demonstrated that macrophage activation stimulates amino acid utilization and glutamine in particular [65, 66]. Here, we will illustrate the involvement of amino acids in macrophage metabolic reprogramming by using two examples—glutamine and arginine.

#### 3.4.1. Glutamine

Glutamine is the most abundant amino acid in the plasma and is internalized in macrophages via the membrane transporter Slc1a5 (solute carrier family 1 member 5) (Figure 2(b)). In macrophages, glutamine is converted into glutamate by the enzyme glutaminase 1 (Gls1). Glutamate is then converted into oxoglutarate to be incorporated in the TCA cycle in the mitochondria [67]. Glutamine is involved in multiple physiological functions including energy supply, nucleotide biosynthesis, and resistance against oxidative stress. At a transcriptional level, glutamine metabolism is highly associated with alternative macrophage polarization upon IL-4 stimulation [55]. Short-term glutamine deprivation of macrophages blunts, at least partially, the increased expression of M2 markers following IL-4-induced activation [55, 68]. This validates the involvement of glutamine metabolism in M2 macrophage metabolic adaptation. Glutamine is essential for the biosynthesis of UDP-GlcNAc which is required for the glycosylation of lectin or mannose receptors that are required for pathogen recognition [55]. Glutamate is also used by M2 macrophages to feed the TCA cycle since more than 30% of the TCA cycle intermediates come from glutamine [55]. Therefore, whereas glutamine is used differently in M1 and M2 macrophages, it appears to be a key resource to support polarization and function of both M1 and M2 macrophages.

Glutaminolysis controls macrophage M2 polarization by regulating epigenetically the transcriptional activity of key genes [68]. Indeed, glutamine supports alternative polarization by favoring Jmjd3- (jumonji domain containing -3-) dependent demethylation on the promoters of M2-associated genes. Interestingly, Jmjd3 controls the activity of the transcription factor IRF4 (interferon regulatory factor 4), and retroviral expression of IRF4 in Jmjd3-deficient macrophages restored normal M2 marker expression [69]. In recent years, monocytes, macrophages, and NK cells have been shown to display a form of memory called trained immunity. Upon infection or vaccination, those cells display long-term changes in their functional programs that allow increased inflammatory response upon secondary response. Glutaminolysis also controls the production of inflammatory cytokines by monocytes in the context of trained immunity, again by modulating gene methylation, a mechanism that seems suited for long-term control of this type of innate memory [70].

#### 3.4.2. Arginine

Arginine is a substrate for nitric oxide synthase (NOS) and plays a key role during NO generation. Three different forms of NOS have been identified: NOS1 (expressed by neurons (nNOS)), NOS2 (inducible and expressed by immune cells (iNOS)), and NOS3 (expressed by endothelial cells (eNOS)). Macrophages express iNOS, and this enzyme is induced after LPS or IFN*γ* (interferon-*γ*) stimulation [71]. Classically activated macrophages convert arginine into NO and citrulline (Figure 2(a)). Interestingly, it was demonstrated recently that NO, through the inhibition of mitochondrial respiration [72, 73], played a key role in the LPS-induced shift from OXPHOS to glycolysis in macrophages [55, 74] (Figure 2(a)). Moreover, the newly generated NO contributes to macrophage killing of intracellular bacteria and host defense against infection. Nevertheless, excessive and uncontrolled NO production leads to cell toxicity and tissue damage. To avoid this scenario, redirecting arginine flux away from iNOS seems to be crucial. In alternatively activated macrophages, arginine is metabolized by the enzyme arginase 1 (Arg1) (Figure 2(b)). This reaction leads to the production of ornithine and urea. Ornithine is subsequently used to generate polyamines and proline. Proline is an important substrate for collagen synthesis and therefore plays a key role in wound healing while polyamines are used during cell proliferation [75, 76]. Thus, during parasite infection, characterized by the generation of M2 macrophages and wound healing, it was expected that macrophage-specific Arg1 deletion will play a detrimental role. Surprisingly, macrophage-specific deletion of Arg1 led to accelerated death during *Schistosoma mansoni* parasite infection [77]. This is not due to an increased susceptibility to infection but rather to excessive liver fibrosis. Interestingly, macrophage arginase 1 expression is required for the control of T cell activation and proliferation [77]. Therefore, arginine plays a key role during macrophage activation and, when it is used by iNOS or Arg1, determines the fate of the cell activation. Of interest, iNOS and Arg1 can be coexpressed and both have their own advantage over arginine processing, with the former having higher affinity while the later showing higher catalytic rate. Therefore, understanding this dual expression and the context in which binding versus activity prevails will certainly shed light on the direction of immune cell responses.

## 4. Conclusion and Future Directions

Metabolism controls key macrophage functions such as phagocytosis and efferocytosis contributing to healthy tissue homeostatic maintenance and protection against infection and inflammation. Diversity of macrophage functions and responses can be modulated through cell-intrinsic metabolic rewiring and/or cell-extrinsic environmental metabolite change. This led to the establishment of an oversimplified but efficient macrophage classification into classically M1 versus alternatively M2 subsets. Nevertheless, this pattern is observed in tissue-resident macrophages at a steady state probably reflecting on their metabolism governed by each specific organ microenvironment. During tissue inflammation, for example, in the case of obese adipose tissue, a switch of macrophage markers towards a proinflammatory phenotype parallels tissue structure remodeling and metabolite availability in the local environment. Therefore, one is interested to control macrophage metabolic demands and maintain their tissue homeostatic role by blocking metabolites leading to a proinflammatory phenotype. This requires better understanding and characterization of tissue macrophage populations and whether conserved metabolic pathways are present and functional in both embryonically and monocyte-derived macrophages. Currently, with the emergence of omics approaches, this aim seems more and more realistic and promises that new metabolic targets used to limit inflammation related to macrophages shall soon be confirmed.

## Abbreviations

Arg1: Arginase 1
Ascl1: Acyl-CoA synthetase long-chain family member 1
ATF3: Activating transcription factor 3
Atgl: Adipose triglyceride lipase
BCAA: Branched-chain amino acids
BCAT-1: Branched-chain aminotransferase 1
*C. albicans*: *Candida albicans*
CCL2: C-C chemokine ligand type 2
CCR2: C-C chemokine receptor type 2
CD36: Cluster of differentiation 36
CXCL12: C-X-C motif chemokine ligand 12
CXCR4: C-X-C motif chemokine receptor 4
DG: Diacylglycerol
ERG240: Leucine analogue
FA: Fatty acid
FAO: Fatty acid oxidation
FDG: 18F-Fluorodeoxyglucose
FFA: Free fatty acid
Gata6: GATA binding protein 6
Gls1: Glutaminase 1
Glut: Glucose transporter
Hif1*α*: Hypoxia-inducible factor 1 subunit alpha
Hsl: Hormone-sensitive lipase
Idh1: Isocitrate dehydrogenase 1
IFN*γ*: Interferon-gamma
IkB*ζ*: Nuclear factor of kappa light polypeptide gene enhancer in B cell inhibitor
IL: Interleukin
IRF4: Interferon regulatory factor 4
Irg1: Aconitate decarboxylase 1
Jmjd3: Jumonji domain containing 3
KEAP1: Kelch-like ECH-associated protein 1
Ldl: Low-density lipoprotein
Ldlr: Ldl receptor
Lipa: Lysosomal acid lipase
*Lipe*: Lipase E
LPS: Lipopolysaccharide
Ly6C: Lymphocyte antigen 6 complex, locus C1
M1: Proinflammatory macrophage
M2: Anti-inflammatory macrophage
MG: Monoacylglycerol
Mgl: Monoacylglycerol lipase
Mtb: Mycobacterium tuberculosis
NK cells: Natural killer cells
NO: Nitric oxide
NOS: Nitric oxide synthase
Nr4a1: Nuclear receptor subfamily 4 group A member 1
Nrf2: Nuclear factor (erythroid-derived 2)-like 2
OXPHOS: Oxidative phosphorylation
PET: Positron emission tomography
PGC1*β*: Peroxisome proliferator-activated receptor gamma coactivator-1 beta
Ppar: Peroxisome proliferator-activated receptor
*Pnpla2*: Patatin-like phospholipase domain containing 2
ROS: Reactive oxygen species
SDH: Succinate dehydrogenase
Slc1a5: Solute carrier family 1 member 5
Spi-C: Transcription factor Spi-C
TCA: Tricarboxylic acid
TG: Triacylglycerol
TLR: Toll-like receptor
TNF*α*: Tumor necrosis factor-alpha
UCP: Uncoupling protein
UDP-GlcNAc: Uridine diphosphate N-acetylglucosamine.
